# The Course of Pain Intensity in Patients Undergoing Herniated Disc Surgery: A 5-Year Longitudinal Observational Study

**DOI:** 10.1371/journal.pone.0156647

**Published:** 2016-05-31

**Authors:** Marie Dorow, Margrit Löbner, Janine Stein, Alexander Pabst, Alexander Konnopka, Hans J. Meisel, Lutz Günther, Jürgen Meixensberger, Katarina Stengler, Hans-Helmut König, Steffi G. Riedel-Heller

**Affiliations:** 1 Institute of Social Medicine, Occupational Health and Public Health, University of Leipzig, Leipzig, Germany; 2 Department of Health Economics and Health Services Research, University Medical Center Hamburg-Eppendorf, Hamburg, Germany; 3 Department of Neurosurgery, Berufsgenossenschaftliche Kliniken Bergmannstrost, Halle (Saale), Germany; 4 Department of Neurosurgery, Klinikum St. Georg gGmbH, Leipzig, Germany; 5 Department of Neurosurgery, University of Leipzig, Leipzig, Germany; 6 Department of Psychiatry and Psychotherapy, University of Leipzig, Leipzig, Germany; The George Washington University, UNITED STATES

## Abstract

**Objectives:**

The aims of this study are to answer the following questions (1) How does the pain intensity of lumbar and cervical disc surgery patients change within a postoperative time frame of 5 years? (2) Which sociodemographic, medical, work-related, and psychological factors are associated with postoperative pain in lumbar and cervical disc surgery patients?

**Methods:**

The baseline survey (T0; n = 534) was conducted 3.6 days (SD 2.48) post-surgery in the form of face-to-face interviews. The follow-up interviews were conducted 3 months (T1; n = 486 patients), 9 months (T2; n = 457), 15 months (T3; n = 438), and 5 years (T4; n = 404) post-surgery. Pain intensity was measured on a numeric rating-scale (NRS 0–100). Estimated changes to and influences on postoperative pain by random effects were accounted by regression models.

**Results:**

Average pain decreased continuously over time in patients with lumbar herniated disc (Wald Chi² = 25.97, p<0.001). In patients with cervical herniated disc a reduction of pain was observed, albeit not significant (Chi² = 7.02, p = 0.135). Two predictors were associated with postoperative pain in lumbar and cervical disc surgery patients: the subjective prognosis of gainful employment (p<0.001) and depression (p<0.001).

**Conclusion:**

In the majority of disc surgery patients, a long-term reduction of pain was observed. Cervical surgery patients seemed to benefit less from surgery than the lumbar surgery patients. A negative subjective prognosis of gainful employment and stronger depressive symptoms were associated with postoperative pain. The findings may promote multimodal rehabilitation concepts including psychological and work-related support.

## Introduction

Degenerative disc disease is commonly accounted as causal in acute and chronic back/leg pain in the general population [1–3]. About 15% of patients with a herniated disc require surgery, because they do not respond to conservative approaches or experience major neurologic losses [4,5]. The main function of surgical treatment is the elimination of pain and associated physical dysfunction [6]. Therefore, the measuring of pain is an important indicator for surgical success.

Studies showed that surgery helps the majority of patients to overcome pain symptoms [7–9], but between 7 and 23% of the operated patients still report severe pain or even experience no pain relief at all [10–14]. While surgical complications may be responsible for persisting symptoms in some patients, these problems do not give an all-embracing explanation for ongoing pain [10,15,16]. Whether a patient benefits sufficiently from surgery or not can most likely be explained by patient characteristics [17]. Research revealed different sociodemographic, medical, occupational and psychological factors that were associated with persistent pain. For example, more intense pain after surgery was related to increasing age [9,11,17–19], stronger preoperative pain [12,20] or a longer duration of preoperative symptoms [11,21]. Relevant work-related factors were a longer time on sick leave [10,12,22] and a reduced ability to work before surgery [12]. Finally, depression [10–13,17,19,23–27], anxiety [20,27,28] as well as dysfunctional cognitive behavioural factors [12,18,27,28] seem to play a particular role in the maintenance of pain.

Even though relevant factors have been discussed, postoperative longitudinal studies are sparse. Hence, little is known about the influence of these factors over time and about postoperative pain fluctuations. The identification of relevant factors may enhance the application of additional support for patients at risk of developing chronic pain syndromes. Chronic pain has been shown to reduce quality of life and results in considerable consumptions of medical resources [29].

Moreover, most studies on postoperative disc problems focus on patients with lumbar disc herniation and do not include patients with cervical disc herniation. In a systematic review on treatment effectiveness for cervical disc herniation Gebremariam et al. [30] suggest that associated prognostic factors should be examined in future studies. Therefore, the following study shall deepen the understanding of postsurgical pain in a sample of both lumbar and cervical disc surgery patients. The *first objective* is to examine the course of pain intensity in lumbar and cervical disc surgery patients over five years of time. The *second objective* is to identify relevant socio-demographic, medical, occupational and psychological predictors for postsurgical pain intensity in lumbar and cervical disc surgery patients over time.

## Materials and Methods

### Study design

**Fig 1:**
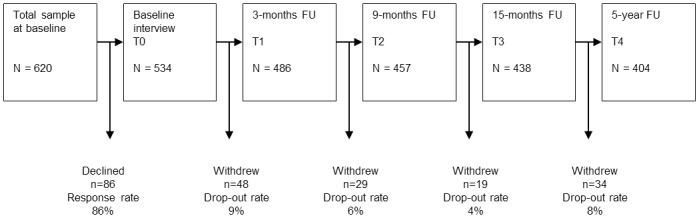
Study design.

This longitudinal cohort study was conducted according to the STROBE statement [31]. The survey included five measuring points (Fig 1). The baseline assessment (T0) was realized 3.6 days (SD 2.48) after disc surgery through face-to-face interviews with trained psychologists in the acute care hospital. In order to observe the patients’ course of pain intensity continuously, the study included three follow-up assessments referring to a short-term, medium-term and early long-term period of time. The first follow-up interview was carried out 3 months (T1), the second follow-up (T2) 9 months and the third follow-up (T3) 15 months after surgery. In addition, we aimed to examine the late long-term effects of surgery. Therefore, a fourth follow-up assessment (T4) was conducted 5 years after surgery. The majority of the follow-up interviews were carried out by telephone through trained psychologists (T1: n = 466; T2: n = 427; T3: n = 399; T4: n = 336). Only a small proportion of the sample could not be reached by telephone and therefore received the paper-pencil version of the interview in form of a written questionnaire (T1: n = 20; T2: n = 30; T3: n = 39; T4: n = 68).

### Sample

Between April 2007 and October 2008, 620 consecutive nucleotomy patients were asked to attend the study. Eighty-six patients declined participation, leading to a number of 534 patients (response rate 86%) included at baseline. Patients were recruited from three different hospitals in Leipzig and Halle (Saale) and had to fulfil the following inclusion criteria:

1. Age between 18 and 55 years,
2. Cervical or lumbar disc herniation as determined by radiological investigation (M50 or M51 diagnosis according to the International Classification of Diseases (ICD-10)),
3. Patients were suitable for rehabilitative care according to the decision of the physician in charge. This decision is based on guidelines of the German health care system on rehabilitation treatment, including the following criteria: First, patients are in need of postsurgical aftercare due to persisting symptoms such as muscular imbalance, persisting pain and/or sensomotoric deficits. Second, patients are physically and mentally able to participate in rehabilitative care. Third, the patients’ functional state is likely to be improved by rehabilitation [32].
4. Patients were able to speak German sufficiently.

### Surgical Procedure

Altogether, n = 422 patients underwent surgery for lumbar disc herniation and n = 112 patients were treated for cervical disc herniation. The majority of patients treated for lumbar disc herniation received standard lumbar microdiscectomy (94%). The remaining 6% were treated with discectomy without use of a microsurgical technique, minimally invasive techniques or other lumbar spine operations. About half of the lumbar disc surgery patients received additional radicular decompression. Most patients with a cervical disc herniation were treated with anterior cervical discectomy (74%). Of these, approximately two thirds received fusion through intervertebral cages. A small amount of patients received posterior cervical discectomy (12%) and in some cervical discectomy patients the surgical access was not specified. Furthermore, most patients were operated on one level (n = 482). The most frequently operated levels were L4/L5 or L5/S1 (n = 376) for the lumbar group and H5/H6 or H6/H7 (n = 87) for the cervical group.

Data on the surgical procedure and number of levels treated could not be collected from all patients, because medical records were either not available or were lacking information. As a consequence, in 42 cases the surgical approach remains unclear. Concerning the number of levels treated, specific data are missing for 21 patients.

### Ethics Statement

The study has received ethics committee approval of the University of Leipzig (Ethik-Kommission an der Medizinischen Fakultät der Universität Leipzig). At the initial contact, participants were verbally informed about the purpose of the study. In addition they received a written study information form. All participants gave written consent to their study participation.

### Variables and Instruments

The data collection consisted of an extensive and wide-ranging set of instruments at each measuring point. All questions asked at follow-up were equivalent for telephone interviews and written surveys.

#### Pain intensity

The primary outcome was the subjective pain intensity. It was assessed at each assessment point using a verbal single-item scale ranging from 0 to 100 (NRS 0–100). The Numeric Rating Scale is a commonly used pain scale and has been studied extensively [33,34]. The choice for a pain rating instrument with many levels was influenced by the fact that this will increase the scale’s ability to detect change. In addition, the NRS can easily be adopted for telephone surveys using the following instruction: "Imagine a scale from 0 to 100. If 0 is no pain and 100 is the worst, please give me a number that indicates the amount of pain you are having today". Consequently, higher scores on the scale indicate stronger pain. Additionally, categories for mild (NRS<30), moderate (NRS≥30 and <70) and severe pain (NRS≥70) were defined based on other studies [11,35].

#### Socio-demographic variables

The following variables were assessed at baseline: age, gender, marital status and highest level of schooling completed.

#### Medical variables

All patients were questioned at baseline about the disc herniation's localization. In addition, the patients were asked at all measuring points whether they had experienced any previous disc herniations and if they had any other chronic diseases. The length of hospital stay was retrieved from the patients' medical records. At T1 patients were asked if they received rehabilitation treatment.

#### Work-related variables

Patients were asked at all times of assessment whether or not they had been employed within the last three months. They were also questioned about their subjective prognosis of gainful employment with the SPE scale [36]. This scale contains three items (score range 0–3). The items relate to (1) the expectancy to stay in a job until retirement based on their current health status, (2) permanent endangerment (subjective) of gainful employment, and (3) current thoughts on applying for an early retirement pension. Higher scores indicate a worse subjective prognosis of gainful employment.

#### Psychological variables

Anxiety and depression were assessed at baseline and all follow-up surveys using the German version of the Hospital Anxiety and Depression Scale (HADS-D) [37]. Higher scores (maximum 21) designate more anxiety and depression, respectively.

### Statistical analysis

Descriptive data are presented as mean±SD or percentages. Baseline differences between the lumbar and the cervical group were calculated via Pearson’s chi-square tests for nominal data and Wilcoxon-Mann-Whitney-tests for ordinal and metric data. Changes in and influences on postoperative pain have been evaluated using multilevel mixed-effects regression models in order to estimate the shape of trajectories of patients over time, while accounting for systematic and random variability [38]. The analysis was performed separately for lumbar and cervical surgery patients. Since pain intensity exhibited a skewed distribution, negative binomial models instead of traditional linear models were conducted. All models included time of assessment (dummy-coded) as well as the socio-demographic, medical, work-related, and psychological covariates as fixed effects on level 1 and a random intercept at level 2 to account for the within-patient heterogeneity. In addition, the consideration of time as random coefficient at level 2 was tested using likelihood-ratio tests. As a result, the effect of time was allowed to vary randomly in the model for patients with lumbar disc herniation. The estimated fixed effects coefficients were transformed to incidence rate ratios (IRRs), representing a percentage change in reported pain intensity per one-unit increase in the predictor. Random effects are presented as variance components with corresponding 95% confidence intervals. In order to reduce the risk of bias sensitivity analyses were conducted, i.e., it was tested whether loss to follow-up significantly influenced outcome estimates. Several methods of replacing missing data were applied, e.g., last value carried forward [LVCF], substitution by mean, inverse probability weighting, and missing imputation. These analyses revealed similar results indicating a negligible influence of loss to follow-up on outcome data. All statistical analyses were performed using the Statistical Package for the Social Sciences 20.0 for Windows (SPSS Inc., Chicago, IL) and Stata 13.1 SE (Stata Corp LP, College Station, TX). In all analyses, a *p*-value below 0.05 was considered statistically significant.

## Results

### Sample characteristics

Table 1 presents the sample characteristics at baseline (T0). The sample included 422 patients with lumbar disc herniation and 112 patients with cervical disc herniation. The total sample consisted of 57% males and had a mean age of 42.4 (SD 8.0) years. The cervical group was approximately 4 years older than the lumbar group. About one third of the patients have had a previous disc herniation before. There was a significant group difference concerning chronic diseases. In the cervical group 55% reported that they had at least one other chronic disease in contrast to 36% in the lumbar group. The mean pain intensity was 31.7 (SD 23.7). Patients in the cervical group had a significantly shorter stay in the acute care hospital and the amount of patients who did not undergo rehabilitation was significantly higher compared to patients in the lumbar group. Most patients were employed within the last 3 months before surgery. The average scores for depression and anxiety were 6.35 (SD 4.3) and 7.43 (SD 4.4), respectively.

**Table 1:** Sample characteristics at baseline.

Variables	Characteristics	Total n (%)	Lumbar n (%)	Cervical n (%)
Sample size T0		534 (100)	422 (79)	112 (21)
Hospital	University Hospital Leipzig	150 (28)	118 (28)	32 (29)
	St. Georg Leipzig	153 (29)	125 (30)	28 (25)
	Bergmannstrost Halle (Saale)	231 (43)	179 (42)	52 (46)
*(a) Socio-demographic variables*				
Gender	Male	306 (57)	247 (59)	59 (53)
	Female	228 (43)	175 (42)	53 (47)
Age (years), mean (SD)	Minimum = 18; Maximum = 55	42.4 (8.0)	41.64 (8.2)	45.34 (6.2)***
Age groups	18–35	111 (21)	106 (25)	5 (5)***
	36–45	212 (40)	166 (39)	46 (41)
	46–55	211 (40)	150 (36)	61 (55)***
Marital status	Single	152 (29)	134 (32)	18 (16)**
	Married	300 (56)	229 (54)	71 (63)
	Separated, divorced	75 (14)	53 (13)	22 (20)
	Widowed	7 (1)	6 (1)	1 (<1)
Highest level of schooling	Up to 9^th^ grade^2^	63 (12)	51 (12)	12 (11)
	10^th^ grade^3^	353 (66)	275 (65)	78 (70)
	Advanced technical college entrance qualification/ University qualification	118 (22)	96 (23)	22 (20)
*(b) Medical variables*				
Previous disc herniation(s)	No	328 (61)	252 (60)	76 (70)
	Yes	187 (35)	154 (37)	33 (30)
Other chronic diseases	No	319 (60)	269 (64)	50 (45)
	Yes	215 (40)	153 (36)	62 (55)***
Pain intensity (NRS), mean (SD)	Minimum = 0; Maximum = 100	31.7 (23.7)	30.9 (23.9)	34.8 (22.6)
	< 30	251 (47)	205 (49)	46 (41)
	≥ 30 and < 70	227 (43)	173 (41)	54 (48)
	≥ 70	55 (10)	43 (10)	12 (11)
Days in hospital, mean (SD)	Minimum = 4; Maximum = 30	8.8 (2.6)	9.0 (2.6)	8.1 (2.7)***
Rehabilitation setting^1^	inpatient rehabilitation	307 (58)	250 (65)	57 (54.8)*
	outpatient rehabilitation	145 (27)	114 (30)	31 (30)
	no rehabilitation	34 (6)	18 (5)	16 (15)***
*(c) Work-related variables*				
Employment within the last 3 months before surgery (other than minijob)	No	108 (20)	85 (20)	23 (21)
	Yes	426 (80)	337 (80)	89 (80)
Subjective prognosis of gainful employment (SPE-scale), mean (SD)	Minimum = 0, Maximum = 3	1.1 (1.0)	1.1 (1.0)	1.2 (1.1)
*(d) Psychological variables*				
Depression (HADS), mean (SD)	Minimum = 0; Maximum = 21	6.35 (4.3)	6.43 (4.4)	6.06 (4.0)
	0–7	357 (67)	279 (66)	78 (70)
	8–14	142 (27)	111 (26)	31 (28)
	15–21	33 (6)	30 (7)	3 (3)
Anxiety (HADS), mean (SD)	Minimum = 0; Maximum = 21	7.43 (4.4)	7.35 (4.4)	7.71 (4.4)
	0–7	304 (58)	242 (58)	62 (55)
	8–14	182 (34)	141 (34)	41 (37)
	15–21	42 (8)	33 (8)	9 (8)

^1^ assessed at T1;

^2^ no school-leaving certificate or secondary general school-leaving certificate;

^3^ intermediate secondary school-leaving certificate

Abbreviations: HADS, Hospital Anxiety and Depression Scale; NRS, Numeric Rating Scale; SD, Standard Deviation

Notes: Pearson`s Chi² test and Wilcoxon-Mann-Whitney-tests were used for pairwise comparison;

* p <.05,

** p <.01,

*** p <.001

### Pain intensity over time

Descriptive statistics showed that pain intensity decreased over time in both groups. The average reduction was larger in the lumbar group with a mean decline from 30.9 (SD 23.9) at T0 to 26.7 (SD 27.0) at T4 compared to a decline from 34.8 (SD 22.6) to 33.4 (SD 29.8) in the cervical group. Concerning the severity of pain, in the lumbar group 10% of the patients reported severe pain at baseline, compared to 14% at T1 and T2, 12% at T3 and 11% at T4. In the cervical group, the amount of patients reporting severe pain was 11% at T0, 15% at T1 and T2, 22% at T3 and 21% at T4.

**Fig 2:**
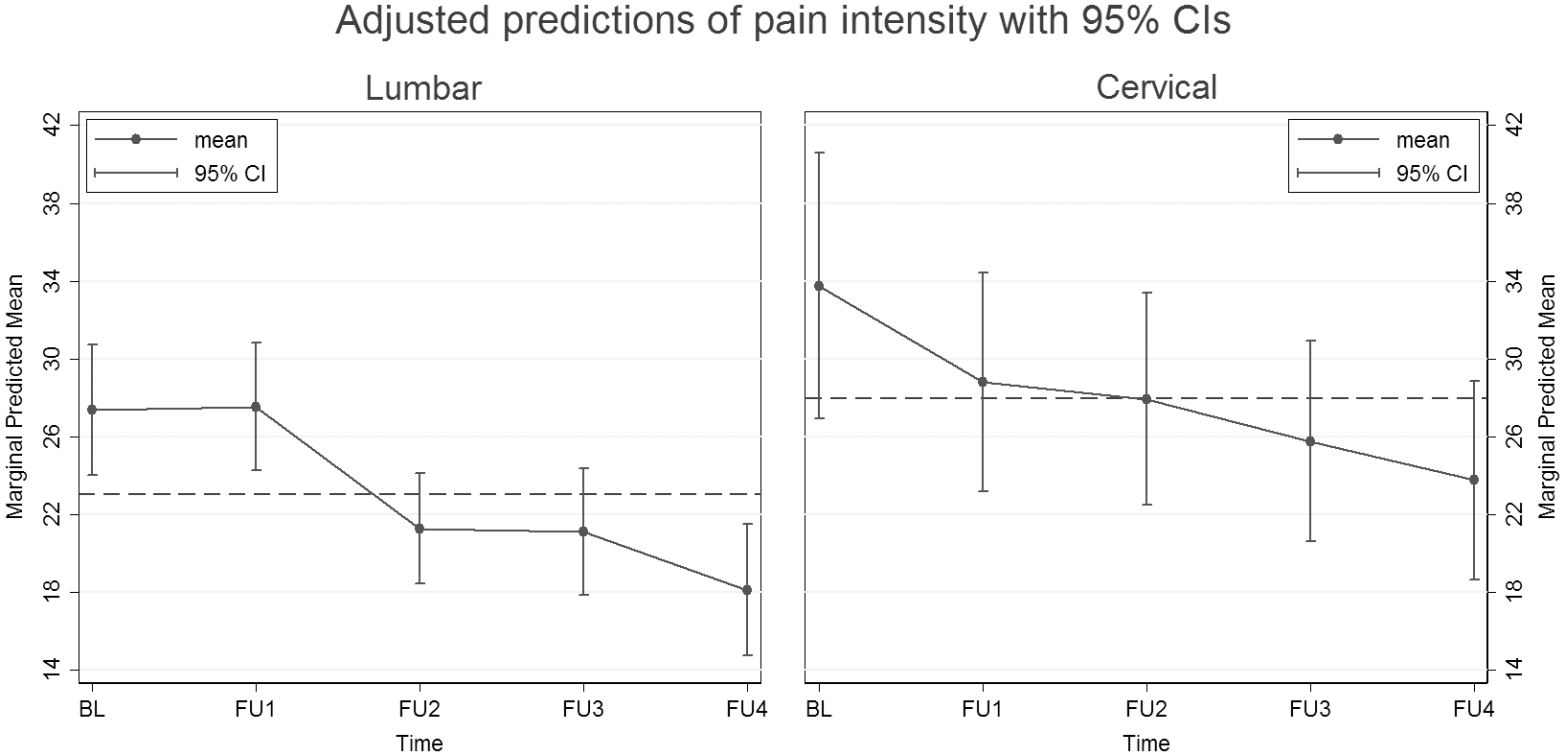
Estimated pain intensity in the course of time (marginal means adjusted for covariates). BL, baseline; CI, confidence interval; FU, follow-up.

Table 2 shows the results of the multilevel mixed-effects negative binomial regression model for the group of patients treated for lumbar herniated disc. Within this model average pain decreased significantly over time (Chi² = 25.97, p<0.001). The comparison of baseline pain with T1 did not reach statistical significance, but pain ratings at T2, T3 and T4 were significantly lower compared to baseline showing a constant decrease. Moreover, pain intensity varied significantly between subjects at T0 (Random Intercept = 0.18, p<0.01) and the courses of pain over time were interindividually different (Random Slope = 0.04, p<0.01). Based on this model the marginal means of pain intensity were calculated for each assessment, holding all other covariates at their means (left side of Fig 2). The estimated marginal pain intensity remained stable from baseline to T1, but then declined continuously from an estimated marginal mean of 27.5 (SE 1.7) at T1 to 18.1 (SE 1.7) at T4.

**Table 2:** Predictors for postoperative pain in patients with lumbar disc herniation (n = 422).

	**IRR**	**95% CI**	**Wald-Chi²**^a^	**p>**C**hi²**
**Fixed effects**				
Time (R: T0)			25.97	<0.001
T1	1.01	0.87, 1.17		
T2	0.78**	0.66, 0.91		
T3	0.77**	0.65, 0.92		
T4	0.66***	0.54, 0.81		
*(a) Socio-demographic*				
Gender (R: female)	0.90	0.77, 1.05		
Age	1.00	0.99, 1.01		
Marital status (R: single)			4.62	0.202
Married	1.18	0.97, 1.44		
Separated/divorced	1.31*	1.01, 1.71		
Widowed	1.28	0.68, 2.41		
Level of schooling (R: up to 9th grade)			1.53	0.465
10th grade	0.84	0.64, 1.11		
Advanced technical college/college/university	0.84	0.61, 1.14		
*(b) Medical*				
Previous disc herniation(s)	0.97	0.92, 1.02		
Other chronic diseases	1.14	1.00, 1.30		
Days in hospital	1.01	0.98, 1.04		
Rehabilitation setting (R: inpatient)			1.72	0.423
outpatient	0.90	0.76, 1.06		
no rehabilitation	0.91	0.64, 1.29		
*(c) Work-related*				
Employment	0.89	0.76, 1.06		
SPE	1.32***	1.23, 1.42		
*(d) Psychological*				
Depression	1.05***	1.03, 1.07		
Anxiety	1.02	1.00, 1.04		
**Random effects variance components**	**Est**	**95% CI**		
Random intercept	0.18**	0.06, 0.29		
Random slope	0.04**	0.02, 0.07		
Log-likelihood	-6973.695			
Number of observations	1662			

*Abbreviations*: CI, confidence interval; Est, estimate; IRR, Incidence Rate Ratio; SPE, subjective prognosis of gainful employment

^a^ Wald Chi^2^ test for testing the joint significance of categorical indicators

* p <.05,

** p <.01,

*** p <.001.

Table 3 shows the results of the mixed-effects regression model for the group of patients treated for cervical herniated disc. Even though showing a slight reduction, average pain did not decrease significantly over time (Chi² = 7.02, p = 0.135). As shown on the right side of Fig 2, the estimated marginal pain intensity was highest at baseline (marginal mean 33.8, SE 3.5) and continuously decreased by an average of 10 points to the lowest level at T4 (marginal mean 23.8, SE 2.6). On average, patients reported higher pain intensity than patients with lumbar disc herniation.

**Table 3:** Predictors for postoperative pain in patients with cervical disc herniation (n = 112).

	**IRR**	**95% CI**	**Wald-Chi²**^a^	**p>Chi²**
**Fixed effects**				
Time (R: T0)			7.02	0.135
T1	0.85	0.66, 1.10		
T2	0.83	0.64, 1.08		
T3	0.76*	0.58, 1.00		
T4	0.70**	0.53, 0.93		
*(a) Socio-demographic*				
Gender (R: female)	0.91	0.72, 1.16		
Age	1.00	0.98, 1.02		
Marital status (R: single)			0.35	0.951
Married	1.08	0.78, 1.51		
Separated/divorced	1.02	0.69, 1.52		
Widowed	1.16	0.33, 4.03		
Level of schooling(R: up to 9th grade)			0.06	0.971
10th grade	0.96	0.65, 1.42		
Advanced technical college/college/university	0.99	0.65, 1.51		
*(b) Medical*				
Previous disc herniation(s)	1.15*	1.03, 1.28		
Other chronic diseases	1.09	0.89, 1.33		
Days in hospital	1.05*	1.01, 1.10		
Rehabilitation setting(R: inpatient)			6.12	0.047
outpatient	0.73*	0.56, 0.96		
no rehabilitation	0.74	0.52, 1.06		
*(c) Work-related*				
Employment	0.99	0.77, 1.28		
SPE	1.32***	1.18, 1.47		
*(d) Psychological*				
Depression	1.08***	1.04, 1.11		
Anxiety	1.00	0.97, 1.04		
**Random effects variance components**	**Est**	**95% CI**		
Random intercept	0.13*	0.03, 0.23		
Log-likelihood	-2016.738			
Number of observations	466			

*Abbreviations*: CI, confidence interval; Est, estimate; IRR, Incidence Rate Ratio; SPE, subjective prognosis of gainful employment

^a^ Wald Chi^2^ test for testing the joint significance of categorical indicators

* p <.05,

** p <.01,

*** p <.001.

### Factors associated with postsurgical pain

In the group of patients treated for lumbar disc herniation, the SPE presented the most influential predictor for pain over time: a one-unit increase in SPE increased the reported postoperative pain intensity by 32% (Table 2). A second significant predictor was depression, i.e., more depressive symptoms predicted higher scores of postsurgical pain (IRR = 1.05, p<0.001).

The same predictors were identified in the group of patients treated for cervical disc herniation: Higher scores on the SPE (IRR = 1.32, p<0.001) and stronger depression (IRR = 1.08, p<0.001) were associated with more intense pain (Table 3). In addition, a higher number of previous disc herniations were associated with stronger postsurgical pain (IRR = 1.15, p<0.05). Further, the number of days in the acute care hospital predicted postsurgical pain (IRR = 1.05, p<0.05) in a way that a longer stay in hospital was associated with stronger postsurgical pain. Finally, the setting of rehabilitation emerged as a significant predictor (Wald Chi² = 6.12, p<0.05). Those patients who underwent outpatient rehabilitation reported less pain than those receiving inpatient rehabilitation.

## Discussion

### Pain intensity in the course of time

In the group of patients treated for lumbar herniated disc pain intensity decreased constantly within 5 years of surgery. The first significant reduction of the average pain intensity assessed at baseline was observed nine months after baseline showing further improvements until the final follow-up. Therefore, in clinical practice, it may be advisable to inform patients that after surgery has been performed it may take up to 9 months until they feel further reductions of pain. The amount of patients with lumbar disc herniation who still suffered severe pain varied between 10 and 14% over time. This goes well in line with other long-term studies with lumbar disc surgery patients using the same cut-off value for severe pain. These showed that the amount of patients who still had severe leg pain [11] or back pain [12] was 8% and 22%, respectively. However, even though a considerable number of patients reported ongoing symptoms in our study, surgery seemed to help the majority of lumbar disc patients to overcome pain symptoms over a long period of time. These findings are supported by other studies showing positive long-term effects of disc surgery [9,11,12,20,23,25,26,39–43]. On the other hand, our long-term results contradict the results of Graver et al. [44] who documented a recurrent increase of pain in lumbar disc surgery patients on a visual analogue scale between 1 year and 7 years after surgery. This indicates the need for further longitudinal postoperative studies in lumbar disc surgery patients.

The group of patients with cervical disc herniation experienced a slight reduction of pain after surgery, albeit not significant. The amount of patients reporting severe pain grew from 11% at baseline to 21% five years later. According to the current literature there is moderate to good evidence for the effectiveness of cervical surgery [45]. However, high-quality RCT studies using validated outcome measures are lacking in this field [30]. In a prospective randomized study [46] short-term outcome for cervical disc surgery was a better predictor for long-term outcome than preoperative data. Again, this highlights the importance of longitudinal studies that investigate pain intensity beyond pre-post comparisons and take into account postoperative pain fluctuations.

Taken together, in both groups the overall estimated marginal pain intensity was mild suggesting that most patients benefitted from surgery. Nevertheless, the question arises why postsurgical pain was significantly reduced over time in patients with lumbar disc herniation but not in patients with cervical disc herniation. A possible answer to this question may lie in the fact that cervical discs provide the mobility of more fragile body areas such as head and neck, while lumbar discs mainly serve as a buffer for mechanical forces resulting from motion. Following this idea, the impairment of head movements may lead to stronger pain per se, but it may also be associated with increased fear of movement which may interfere with recovery. In addition, cervical disc herniation is less frequent than lumbar disc herniation and affected patients have a higher risk of developing spinal cord injuries. This in turn may lead to stronger insecurity and health concerns which may be expressed through higher pain ratings. Future research should deepen the understanding of pain-related outcome differences between lumbar and cervical disc surgery patients.

### Predictors for postsurgical pain

Relevant associations with postsurgical pain were identified in both groups. The SPE and depression emerged as influential predictors on postoperative pain in both lumbar and cervical disc surgery patients.

Patients with a worse SPE reported significantly stronger pain. This finding is supported by publications from Johansson et al. [41] and Junge et al. [12] who found that a stronger wish to retire and a lower perceived chance to regain work were significantly related to pain. Stengler et al. [47] found out that a negative SPE was associated with a worse quality of life in disc surgery patients.

A relation between depression and pain in disc surgery patients was reported in numerous studies [10–13,17,19,23–27,48,49]. These empirical findings are in line with theoretical conceptions on pain which all agree that pain perception involves not only sensory but also behavioural, affective and cognitive components [50]. One such model is the biopsychosocial model of chronic pain by Hasenbring [15]. In this model, depression represents a major factor for pain chronification in lumbar syndromes and coping reactions play an important role for physiological changes such as intradiscal pressure.

In addition to these factors, in the group of patients with cervical disc herniation, the number of previous disc herniations, the number of days in hospital and the rehabilitation setting influenced the patients’ pain ratings. A longer hospital stay may be the result of surgical complications or unsatisfactory recovery leading to more intense pain. Recurrent herniations may be an indication for a more complicated course of the disease and may indicate a stronger degree of degeneration. This in turn is associated with worse physical health [51] and may therefore explain higher pain intensity ratings. Patients receiving outpatient rehabilitation treatment reported less pain than patients undergoing inpatient rehabilitation. This may be due to the fact that patients with a better health status preferred an outpatient rehabilitation setting, while those experiencing stronger pain after surgery were more likely to choose inpatient rehabilitation.

### Limitations

For the benefit of a larger sample size the baseline assessment was carried out after surgery as the presurgical time frame was too short to reach all consecutive patients. Hence, time of assessment may explain why significant changes in the short-term period did not become obvious. A second limitation is that the patients’ pain ratings are subjective in nature and accurate measures must rely on self-reports. On the other hand, the NRS is a frequently used scale for pain intensity and shows good evidence of reliability and validity [33]. Moreover, the surgical procedure and number of levels treated may have influenced the patients’ postoperative pain intensity. As this information was not available for all patients, it could not be included in our analyses. The lack of availability of this data is an inherent weakness in the study design. Finally, the findings in this study refer to a German patient population and may therefore not apply to other countries.

## Conclusion

The objectives of this study were (1) to examine the course of pain intensity in lumbar and cervical disc surgery patients over 5 years of time and (2) to identify independent sociodemographic, medical, occupational and psychological predictors for pain.

In the majority of disc surgery patients, a long-term reduction in pain was observed. However, the cervical surgery patients seemed to profit less from surgery than the lumbar surgery patients. In the long-term a considerable number of patients still reported high levels of pain. The most important risk factors for postsurgical pain were a negative SPE and depression. The results promote a multimodal treatment setting including psychological and vocational support for patients at risk of pain chronification. Finally, the present findings may be taken into consideration when it comes to developing screening instruments to identify patients at risk as well as to provide a better patient selection for surgical approaches.

## Supporting Information

S1 Checklist STROBE Checklist. (DOC)
